# Does Foveal Position Relative to the Optic Disc Affect Optical Coherence Tomography Measurements in Glaucoma?

**DOI:** 10.4274/tjo.56254

**Published:** 2018-09-04

**Authors:** Zerrin Tuncer, Mitat Altuğ

**Affiliations:** 1 Göz Vakfı, Bayrampaşa Eye Hospital, Ophthalmology Clinic, İstanbul, Turkey

**Keywords:** Optical coherence tomography, Bruch’s membrane opening, minimum rim width, optic disc, glaucoma

## Abstract

**Objectives:**

To determine interindividual variability in the angle between the anatomic axis connecting the fovea and optic disc center and the horizontal meridian using spectral domain optical coherence tomography (OCT).

**Materials and Methods:**

A total of 260 eyes of 133 subjects (81 women, 52 men) with glaucoma or suspected glaucoma were included in the study retrospectively. Fovea-disc angle (FoDi angle) measurements, determined as the angle between the horizontal meridian passing through the Bruch’s membrane opening (BMO) center and the line connecting the fovea and BMO center, were recorded from spectral domain-OCT scans performed by the same investigator. FoDi angle was defined as negative if the fovea was located below the horizontal meridian through the BMO center and positive if the fovea was located above it.

**Results:**

The mean age of the participants was 56.5±14.6 years (27-83 years). The mean FoDi angle was -6.43±4.96° (range: -24.40° to +11.60°). Absolute deviation of the fovea BMO axis from the horizontal axis was 0-5° in 83 eyes (31.92%), 5-10° in 124 eyes (47.69%), 10-15° in 41 eyes (15.76%), 15-20° in 10 eyes (3.84%), and greater than 20° in 2 eyes (0.79%).

**Conclusion:**

Most OCT devices currently used in the treatment and follow-up of glaucoma patients provide peripapillary retinal nerve fiber layer (RNFL) thickness measurements that are made based on a clinical axis in reference to the horizontal meridian passing through the optic disc center. The results of our study reveal interindividual variation in FoDi angle as well as intraindividual differences in FoDi angle between fellow eyes in the same individual. Disparity between clinical and anatomic quadrants could impact RNFL thickness measurements, which may lead to errors in the diagnosis of glaucoma.

## Introduction

Glaucoma is among the leading causes of blindness worldwide.^1^ Optical coherence tomography (OCT) parameters are reported to be more sensitive than visual field parameters for the early diagnosis of glaucoma.^2^ OCT provides objective, measurable, and reproducible values pertaining to peripapillary retinal nerve fiber layer (RNFL) thickness and the optic nerve head (ONH), making it an important tool for early diagnosis and progression monitoring in glaucoma.^3^

**Figure 1:**
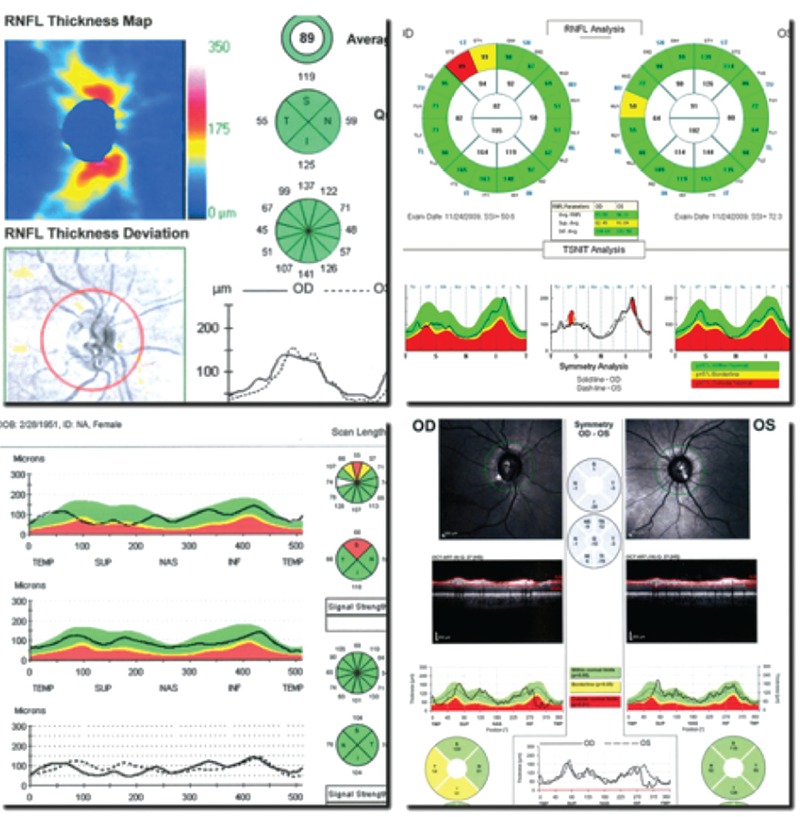
Measurements made in various optical coherence tomography devices based on a 0-180° horizontal meridian RFNL: Retinal nerve fiber layer, OD: Right eye, OS: Left eye

Following the first publication on OCT over 25 years ago, various studies have shown that OCT is an important technological development for glaucoma diagnosis.^4,5,6,7,8^ The use of spectral-domain OCT (SD-OCT) technology enables the acquisition of more detailed information with higher image resolution and scanning speed compared to time-domain devices (TD-OCT).^9,10^ Nevertheless, the value and reliability of OCT for early diagnosis and follow-up in clinical practice are still a subject of debate.^11,12,13^ With the widely used SD-OCT devices, the optic disc margins are estimated clinically and RNFL thickness measurements are made by accepting the 0-180° horizontal line passing through the ONH center as the horizontal meridian (Figure 1). In addition, achieving the same head position and measuring through exactly the same points in repeated scans done to assess progression is entirely a matter of chance, which raises questions about reliability.

**Figure 2:**
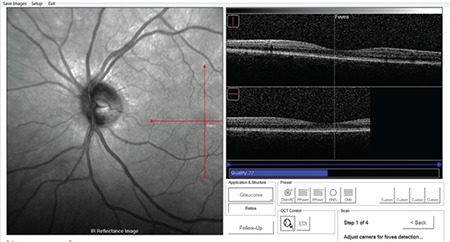
Automatic fovea detection in the fovea-disc software

**Figure 3:**
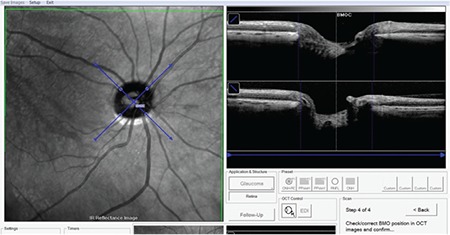
Determination of optic disc margins based on the Bruch’s membrane opening in the fovea-disc software

**Figure 4:**
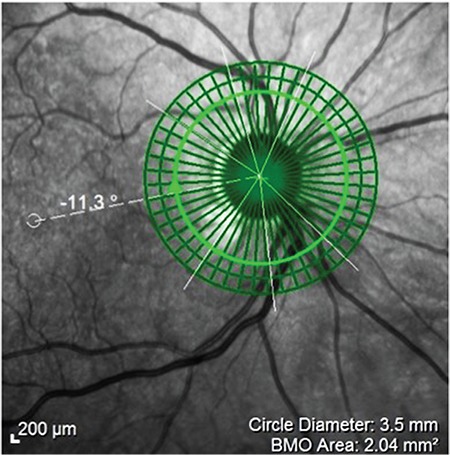
Retinal nerve fiber layer thickness measurement with the fovea-disc software BMO: Bruch’s membrane opening

In the March 2015 update to the SD-OCT (Spectralis; Heidelberg Engineering GmbH, Heidelberg, Germany) device, fovea-disc (FoDi) alignment software was added with version 6.0. When measuring RNFL thickness with this new software, the fovea is first identified automatically (Figure 2), then the optic disc margins are automatically detected (Figure 3), and RNFL thickness is measured based on quadrants determined using the anatomic axis connecting the fovea and ONH center (Figure 4). As the same axis is used when preparing the database, the results obtained from data analysis are more accurate and rotational errors are eliminated. In addition, the FoDi system enables comparison of the same points in serial measurements of the same patient, allowing a more accurate assessment of progression.

The aim of this study was to utilize the FoDi software to investigate interindividual differences in the angle between the axis connecting the fovea center and optic disc center and the horizontal meridian through the OHN center among patients with suspected or diagnosed glaucoma. Considering that RNFL measurements with other devices are made using quadrants based on a standard 0-180° horizontal axis, we wanted to bring attention to the possible effect the discrepancy between these axes may have on diagnostic evaluation.

## Materials and Methods

Two hundred sixty eyes of 133 patients (81 females, 52 males) who were diagnosed with glaucoma or were being followed for suspected glaucoma at our hospital were retrospectively included in the study. Patients having a best corrected visual acuity of 20/40 or better, refractive errors with a spherical equivalent of less than ±5.00 diopter, and no macular pathology were included in the study. Patients with a history of previous eye trauma or surgery, peripapillary atrophy, and tilted discs were not included. Data from previous routine ophthalmological examinations, including visual acuity measurements, refractive error assessment, slit-lamp and fundus examinations, and intraocular pressure measurements with Goldmann applanation tonometry, were recorded.

Prior to OCT, all patients’ pupils were dilated with 1% tropicamide. Patients were asked to look at the internal foveal fixation point during measurement. All RNFL thickness measurements were obtained by the same experienced physician (T.Z.) using the glaucoma module (software version 6.0f) of the SD-OCT device (Spectralis; Heidelberg Engineering GmbH, Heidelberg, Germany). Measurements were repeated for eyes with measurement quality less than 15. The angles between the axis connecting the fovea center and optic disc center and the horizontal axis passing through the disc center (FoDi angle, automatically provided in the OCT output) were recorded. In the OCT output, angles were recorded as negative values if the fovea center was below the horizontal axis through the ONH center and as positive values if the fovea center was above the horizontal axis. Absolute deviation values between the anatomic axis and horizontal axis (irrespective of positive/negative direction of FoDi angle) were also recorded.

### Statistical Analysis

SPSS software (version 22.0, SPSS, Inc.) was used for statistical analyses. The one-sample Kolmogorov-Smirnov test was used to assess the normality of FoDi angle distribution and the distribution of absolute misalignment angle according to the horizontal axis.

## Results

The patients ranged in age from 27 to 83 years, with a mean age of 56.5±14.6 years.

FoDi angles ranged between -24.40° and +11.60°, and the mean value taking into account positive and negative direction was -6.43±4.96°. Of the 260 eyes in the study, FoDi angle was negative in 243 eyes (93.46%) and positive in 16 eyes (6.15%). The anatomical axis and the horizontal axis were on the same plane in only 1 eye. According to the one-sample Kolmogorov-Smirnov test, FoDi angle distribution was normal (p=0.002) (Graphic 1). Analysis of deviations between 0° and 24.4° without regard to positive or negative sign yielded a mean absolute deviation of 7.14±3.95° between the anatomic axis and the horizontal axis. The distribution of absolute deviation values was also normal according to one-sample Kolmogorov-Smirnov test (p=0.2) (Graphic 2).

Deviation from the horizontal axis was 0-5° in 83 eyes (31.92%), 5-10° in 124 eyes (47.69%), 10-15° in 41 eyes (15.76%), 15-20° in 10 eyes (3.84%), and greater than 20° in 2 eyes (0.79%).

## Discussion

Morphological changes in glaucoma are detected by assessing the optic nerve head and RNFL. Visual field testing, which is also used in glaucoma diagnosis and follow-up, is a subjective method. OCT emerged in the early 1990s but was more widely used after the introduction of the TD Stratus OCT (Carl Zeiss Meditec) in 2001. The production of the Optovue OCT (Optovue Inc., Fremont, California, USA) device in 2006 and the Cirrus OCT (Carl Zeiss Meditec) device in 2007 using SD-OCT technology enabled more detailed examination of the optic nerve head in addition to the RNFL, and the use of OCT became increasingly widespread. Improvements in SD-OCT technology have enabled the visualization of various anatomic features in the assessment of the ONH which were not available until recently.

**Figure 5:**
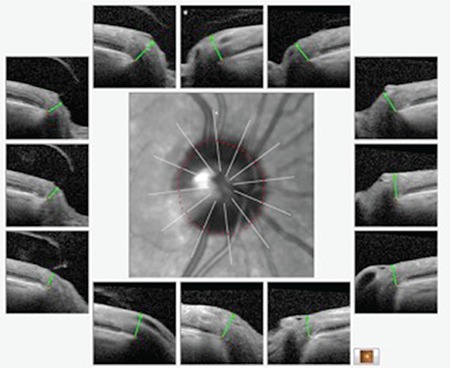
Determination of optic disc margins based on Bruch’s membrane opening in the fovea-disc software

**Figure 6:**
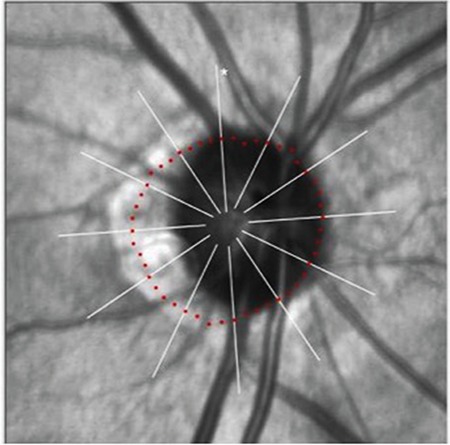
Anatomic disc margins according to Bruch’s membrane openingminimum rim width

The glaucoma module of the Spectralis SD-OCT device (software version 6.0; Heidelberg Engineering GmbH, Heidelberg, Germany) evaluates the neuroretinal rim by automatically determining the shortest distance from the inner rim of the BMO to the internal limiting membrane as a reference and measuring RNFL thickness (Figure 5). The BMO minimum rim width (BMO-MRW) is now defined as a new parameter for the diagnosis of glaucoma. Determining MRW in this way is said to result in the most geometrically accurate measurement compared to scanning methods that have been in use since the introduction of OCT.^7,14^ Kook et al.^5^ reported that in the ONH, BMO-MRW represents the outer margin of the neuroretinal rim and is an anatomically definitive structure that allows neuroretinal rim measurements. When making measurements with standard OCT devices, the optic disc margins are determined by clinical estimation, which is very error-prone (Figure 6). In their study, Helvacioglu et al.^15^ emphasized the importance of correctly positioning the 3.4 mm circle when measuring RNFL thickness. Reis et al.^20^ stated in their study that due to clinically invisible extensions of the Bruch membrane and sectoral differences, the clinically observable disc margin is not the true disc margin. Numerous studies have emphasized the importance in preperimetric glaucoma of OCT measurements of RNFL thickness taking into account BMO.^17,18,19,20^

The position of the fovea relative to the optic disc center has recently attracted the attention of some glaucoma researchers and new software has been developed. This is because measurements based on a horizontal meridian do not allow measurements at the same points when monitoring progression, and errors related to head position can lead to misleading results.

**Figure 7:**
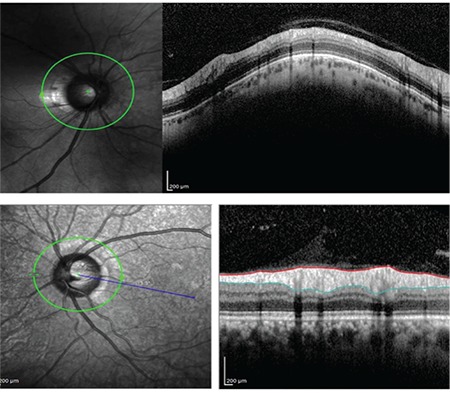
Retinal nerve fiber layer thickness measurement before and after foveadisc software

The FoDi software add-on found in the glaucoma module of the Heidelberg SD-OCT device automatically provides values for the ocular torsion angle. With the Anatomic Positioning System (APS) in the glaucoma module of Heidelberg SD-OCT, disc margins can be detected automatically based on BMO.^7,9^ Using a device with this updated software, the line connecting the center of the optic disc margins automatically determined based on BMO (optic disc center) and the automatically determined fovea center was identified as the anatomical axis. The superior, inferior, nasal, and temporal quadrants are determined based on this axis (Figure 7). We believe this increased the accuracy of the RNFL thickness measurement values we obtained in this study.

There are various studies in the literature concerning the importance of FoDi alignment in the measurement of peripapillary RNFL thickness. In a study analyzing Stratus OCT data of 94 healthy individuals, Vizzeri et al.^9^ reported positive linear relationships between signal strength and horizontal deviation and mean RNFL thickness. Similarly, Chauhan and Burgoyn^21^ stated that the axis between the fovea and center of the BMO-based optic disc must be used in neuroretinal rim and peripapillary RNFL thickness measurements, otherwise the diagnostic sensitivity of SD-OCT measurements may be reduced.

Helvacioglu et al.^15^ measured RNFL thickness in 42 eyes of 21 patients using Optovue SD-OCT and compared measurements made by perfectly centering to the ONH with measurements that were misaligned to the 4 quadrants (superior, inferior, nasal, temporal), and found that misalignment led to falsely low thickness measurements in that quadrant and higher thickness measurements in the opposite quadrant. Similarly, in their study including 222 eyes of 222 patients with ocular hypertension or glaucoma, He et al.^22^ emphasized the importance of foveal position relative to the optic disc for normal RNFL thickness distribution. In another study including 164 eyes of 164 non-glaucomatous myopic patients, Choi et al.^23^ reported that in eyes with a more negative FoDi angle, the peripapillary RNFL thickness profile was thinner in the superior quadrant and thicker in the inferior quadrant.

Contrary to these studies, Mwanza et al.^24^ reported that FoDi alignment had no marked effect on RNFL thickness measurements in their Cirrus HD OCT study including 282 healthy (normative database), 46 non-glaucomatous myopic, and 86 glaucomatous individuals.

**Figure 8:**
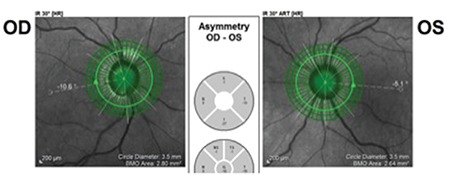
Fovea-disc angle differences between fellow eyes of the same patient OD: Right eye, OS: Left eye

The aim of the present study was to emphasize the interindividual variation in FoDi angle, which affects peripapillary RNFL thickness, and to suggest that this variability may impact RNFL thickness measurements and ultimately lead to inaccuracy in glaucoma diagnosis. There are also various studies in the literature attempting to determine this difference. Reis et al.^25^ found in their study that the optic disc margin tissue is variable and the angle between the horizontal axis and the axis connecting the fovea and the optic disc center varies between individuals and even between fellow eyes of the same individual. We also observed this intraindividual difference in FoDi angle in all of our patients (Figure 8).

**Figure 9:**
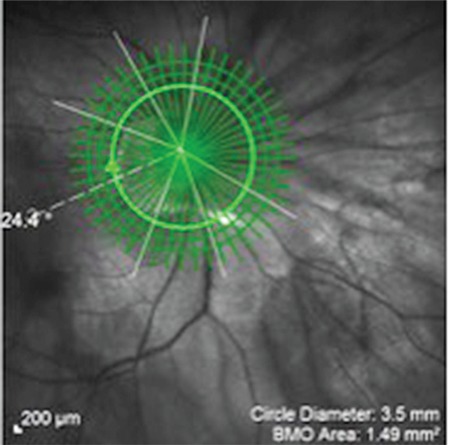
Fovea-disc angle of 24.4°

In previous studies, mean FoDi angle values range between -5.6° and -7.7° and the FoDi distribution is between -17° (the fovea is 17° below the disc) and +7° (the fovea is 7° above the disc).^19,26,27,28^ Consistent with the literature, the range of FoDi angles in our patients was -24.4° to +11.6° and the mean value was -6.43±4.96°. The range of absolute deviation angles between the anatomical axis and the horizontal axis was 0-24.4°, and the mean absolute deviation was 7.14±3.95°. In our study, the anatomical axis and the horizontal axis were on the same plane in only 1 of 260 eyes. We observed FoDi angles greater than 10° in 20.39% of the eyes (53 eyes) and greater than 20° in 0.79% of the eyes (2 eyes) (Figure 9).

## Conclusion

In conclusion, we believe that the disparity between the anatomic axis and horizontal axis observed among patients with diagnosed or suspected glaucoma in our study may affect RNFL thickness measurements and lead to errors in early glaucoma diagnosis. Comparative studies on larger patient series are needed for more definitive results.

## Figures and Tables

**Graphic 1:**
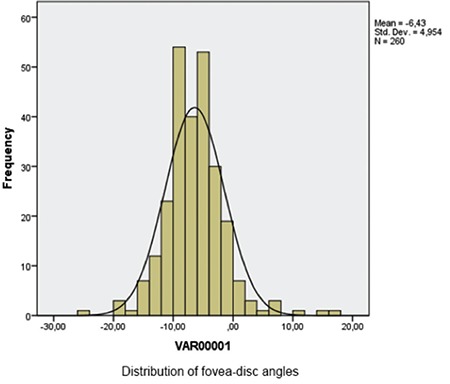
Distribution of fovea-disc angles between -24.40° and +11.60° (x axis: fovea-disc angles in degrees; y axis: number of eyes)

**Graphic 2:**
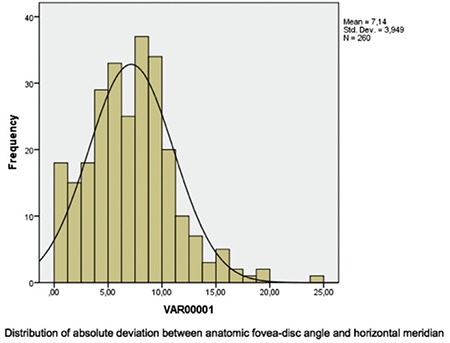
Distribution of absolute deviation of anatomic fovea-disc angle from horizontal meridian, ranging from 0° to 24.4° (x axis: absolute deviation in degrees; y axis: number of eyes)
